# Primary Health Care: An Important Approach for Health Sector, Missed in Iran’s Health System Evolution Plan

**Published:** 2017-09

**Authors:** Masoud BEHZADIFAR, Masood TAHERI MIRGHAED, Aidin ARYANKHESAL

**Affiliations:** 1. Health Management and Economics Research Center, Iran University of Medical Sciences, Tehran, Iran; 2. Dept. of Public Health, Faculty of Health and Nutrition, Lorestan University of Medical Sciences, Khorramabad, Iran; 3. Dept. of Health Services Management, School of Health Management and Information Sciences, Iran University of Medical Sciences, Tehran, Iran

## Dear Editor-in-Chief

One of the most effective and valuable principles to promote health in all countries has been adoption of Primary Health Care (PHC) as a fundamental strategy. In Sept. 1978, an international conference was held in Alma-Ata, whose great achievement was declaring PHC as a roadmap for “Health for All” by the year 2000 (HFA 2000). Indeed, as a new approach beyond the traditional system of health care, PHC insisted on justice in the delivery and distribution of services in the health sector (1). Hence, PHC needs a reasonable development in the health sector as well as in economic and social sections in order to facilitate individuals’, families’ and communities’ access to basic but necessary health services. The first purpose of PHC was achieving a level of physical, psychological and social well-being that people can make fair interaction with their surrounding world. In fact, PHC is the cornerstone of health systems worldwide (2).

The PHC seeks increasing equity in the health sector, reducing public spending, increasing universal coverage of health services, reducing deficiencies in health status and, above all, involving people in the field of health promotion and delivery of care. World Health Organization (WHO) in its 2008 Health Report entitled “Primary health care, now more than ever” reaffirmed the importance of PHC. However, a large share of the financial resources is paid for the secondary healthcare, while the PHC can reduce up to 70% of the global burden of disease with much less cost. The report necessitates health systems to take four steps towards fulfilling the PHC goals, including (i) universal coverage of people based on their needs, with no attention to ability to pay, (ii) making health systems more people-centered, so that healthcare is more responsive to the social and local changes, (iii) integrating public health with primary health through public policy making, and (iv) making the governments more reliable through negotiation-based leadership (3).

As in many countries, Iran used to deliver comprehensive PHC and was one of the first followers of WHO’s model in this field. After the Islamic Revolution, the first PHC programs in Iran were conducted. Qualified local women and men were educated as “Behvarz”, located at “health houses”, responsible for the implementation of PHC programs in rural areas of Iran. Such simple and inexpensive program, along with the special attention of health policymakers and political leaders made many achievements in the health sector (4). However, the program was not updated based on the increasing needs and expectations for certain reasons.

Trying to copy Iran’s PHC model, many nations made valuable initiatives to achieve and maintain the PHC goals, (5) but unfair distribution of financial resources in Iran (6) and tendency of Ministry of Health and Medical Education (MoHME) to specialized medical education rather than preventive, resulted gradually in plans and reforms through which the PHC programs substituted with advanced treatment and curative services (secondary health care), because the latter provides more money than the first to the health sector and caregivers (7). Such gap between primary and secondary care increased in 2014 when Iran’s MoHME made a series of changes, known as Health System Evolution Plan (HSEP). Although the plan had some positive impacts (8), it focused generally on the secondary care through its eight strategies of reduction of patients’ payments, promotion of natural delivery, improvement of hotel services in hospitals, maintenance of doctors in less developed regions, reasonable healthcare charges, full-time (24/7) presence of consultant doctors in hospitals, improvement of the quality of consultant visits, and improvement of emergency services (9). Besides, HSEP’s excessive focus on the secondary healthcare, it led to rise of health care charges, under the strategy of “making charges reasonable” which increased the costs of the health sector, payable from government’s pocket to physicians and hospitals. As a result, other sectors’ budget such as primary health care and rehabilitation were marginalized even more (10).

PHC strategy has changed global attitudes to health issues around the world. On the contrary, Iran’s current HSEP seems to break most its PHC-based strategies, trapped in, and overwhelmed by costly and rather inappropriate curative care. This can be an end to the PHC in Iran if the authorities do not make prompt and necessary actions to get the systems focus back on primary health care.
